# Cytoprotective effects of fermented oyster extracts against oxidative stress-induced DNA damage and apoptosis through activation of the Nrf2/HO-1 signaling pathway in MC3T3-E1 osteoblasts

**DOI:** 10.17179/excli2020-2376

**Published:** 2020-08-04

**Authors:** Cheol Park, Hyesook Lee, Min Ho Han, Jin-Woo Jeong, Sung Ok Kim, Soon-Jeong Jeong, Bae-Jin Lee, Gi-Young Kim, Eui Kyun Park, You-Jin Jeon, Yung Hyun Choi

**Affiliations:** 1 Division of Basic Sciences, College of Liberal Studies, Dong?eui University, Busan, Republic of Korea; 2 Anti‐Aging Research Center, Dong‐eui University, Busan, Republic of Korea; 3 Department of Biochemistry, Dong‐eui University College of Korean Medicine, Busan, Republic of Korea; 4 National Marine Biodiversity Institute of Korea, Seocheon, Republic of Korea; 5 Freshwater Bioresources Utilization Bureau, Nakdonggang National Institute of Biological Resources, Sangju, Republic of Korea; 6 Department of Food Science and Biotechnology, College of Engineering, Kyungsung University, Busan, Republic of Korea; 7 Department of Dental Hygiene, College of Health Science, Youngsan University, Yangsan, Republic of Korea; 8 Ocean Fisheries & Biology Center, Marine Bioprocess Co., Ltd., Busan, Republic of Korea; 9 Department of Marine Life Science, Jeju National University, Jeju, Republic of Korea; 10 Department of Oral Pathology and Regenerative Medicine, School of Dentistry, Kyungpook National University, Daegu, Republic of Korea

**Keywords:** fermented oyster extract, ROS, DNA damage, apoptosis, Nrf2/HO-1

## Abstract

Osteoblast damage by oxidative stress has been recognized as a cause of bone-related disease, including osteoporosis. Recently, we reported that fermented Pacific oyster (*Crassostrea gigas*) extracts (FO) inhibited osteoclastogenesis and osteoporosis, while promoting osteogenesis. However, since the beneficial potential of FO on osteoblasts is not well known, in the present study, we investigated the cytoprotective effect of FO against oxidative stress in MC3T3-E1 osteoblasts. Our results demonstrated that FO inhibited hydrogen peroxide (H_2_O_2_)-induced DNA damage and cytotoxicity through the rescue of mitochondrial function by blocking abnormal ROS accumulation. FO also prevented apoptosis by suppressing loss of mitochondrial membrane potential and cytosolic release of cytochrome *c*, decreasing the rate of Bax/Bcl-2 expression and reducing the activity of caspase-9 and caspase-3 in H_2_O_2_-stimulated MC3T3-E1 osteoblasts, suggesting that FO protected MC3T3-E1 osteoblasts from the induction of caspase dependent- and mitochondria-mediated apoptosis by oxidative stress. In addition, FO markedly promoted the activation of nuclear factor-erythroid-2-related factor 2 (Nrf2), which was associated with the enhanced expression of heme oxygenase-1 (HO-1). However, inhibiting the expression of HO-1 by artificially blocking the expression of Nrf2 using siRNA significantly eliminated the protective effect of FO, indicating that FO activates the Nrf2/HO-1 signaling pathway in MC3T3-E1 osteoblasts to protect against oxidative stress. Based on the present data, FO is thought to be useful as a potential therapeutic agent for the inhibition of oxidative stress in osteoblasts.

## Introduction

Osteoporosis is a common bone disease that seriously affects the quality of life of patients. It is well known that the imbalance between bone resorption and formation is a critical cause of osteoporosis, especially in older people (Montaner Ramón, 2019[45]; Rachner et al., 2011[48]). Over-activation of osteoclasts is accepted as the main etiology of osteoporosis, but osteoblasts are important for osteogenesis and bone remodeling, because new bone formation is basically dependent on osteoblasts. Therefore, the removal of factors that interfere with osteoblast function together with promotion of the proliferation and differentiation of osteoblast precursor cells may be an appropriate strategy for treating osteoporosis (Gennari et al., 2020[18]; Li et al., 2018[41]). Several previous studies have shown that oxidative stress due to excessive generation of reactive oxygen species (ROS) or destruction between the antioxidant and the oxidative defense systems is an important factor in the development and progression of osteoporosis (Schröder, 2019[50]; An et al., 2016[2]). Under physiological conditions, ROS act as intracellular signal transduction molecules, but excessive accumulation of ROS promotes the proliferation and differentiation of osteoclasts by increasing the expression of receptor activator of NF-kappaB ligand (RANKL) (Agidigbi and Kim, 2019[1]; Kim et al., 2010[35]). On the other hand, high ROS levels interfere with redox equilibrium in osteoblasts, inhibit their proliferation and differentiation, promote osteoblast damage and apoptosis, and further reduce bone formation and mineralization (Schröder, 2019[50]; Domazetovic et al., 2017[12]). These observations indicate that it is essential to up-regulate the oxidative defense system to eliminate ROS production, especially in osteoblasts, to suppress bone loss and prevent osteoporosis.

Currently, many studies for protecting cells from oxidative stress-induced damage have focused on the control of key transcription factors. One of them is nuclear factor-erythroid 2-related factor 2 (Nrf2), which regulates the production of antioxidant enzymes for defense against oxidative stress-mediated disorders, including osteoporosis (Xu et al., 2020[60]; Tian et al., 2019[53]; Kim et al., 2014[34]). Nrf2 is present in the cytoplasm with binding to its negative regulator, Kelch-like ECH-associated protein-1 (Keap1), under homeostatic conditions, and degraded *via* the ubiquitin proteasome system. However, when cells are exposed to oxidative stress, Nrf2 escapes from Keap1 and is translocated into the cell nucleus to promote the transcriptional activity of the response genes of the antioxidant response element (ARE) (Shaw and Chattopadhyay, 2020[51]; Fetoni et al., 2019[15]). Among inducible phase II enzymes regulated at the level of transcription by ARE, heme oxygenase-1 (HO-1) breaks down heme into carbon monoxide, free iron, and biliverdin. Since biliverdin is further broken down into bilirubin, which has antioxidant properties, HO-1 plays a potentially important role in antioxidant defense and bone absorption, as well as iron homeostasis (Yu et al., 2018[63]; Kanzaki et al., 2016[31]; Bonelli et al., 2012[4]). All of this evidence suggests that the application of antioxidants capable of activating the Nrf2/HO-1 signaling pathway could be adopted as a promising therapeutic strategy for the prevention and treatment of osteoporosis.

In recent years, the use of food resources and natural compounds as an alternative therapy for the prevention and treatment of various diseases, including osteoporosis, has widely increased. Among marine organisms, oysters are one of the edible shellfish that have long been widely used in industrial resources, as well as valuable food, in many countries, including Korea. Oyster shells have traditionally been widely used to improve bone tissue regeneration, because they are rich in calcium, and contain a variety of minerals. Recently, various oyster extracts have been reported to have strong antioxidant effects. For example, Umayaparvathi et al. (2014[54]) have reported that the hydrolysates of oyster (*Saccostrea cucullata*) protein have highly free radical-scavenging ability. It has also been suggested that phenolic compounds isolated from the Pacific oyster (*Crassostrea gigas*) can prevent the apoptosis of hepatocytes by oxidative stress, and are useful for the prevention and treatment of liver diseases related to oxidative processes (Watanabe et al., 2012[57][56], Fuda et al., 2015[17]). In addition, oyster (*C. hongkongensis*) polysaccharides have been shown to improve intestinal mucositis by promoting intestinal cell proliferation, partially preventing mucosal damage, and reducing inflammation (Cai et al., 2018[7]), and have a protective effect in intestinal cells against hydrogen peroxide (H_2_O_2_)-mediated oxidative stress (Cai et al., 2019[6]). Furthermore, polysaccharides isolated from *Ostrea rivularis* have been shown to reduce H_2_O_2_-induced ROS production, and prevent apoptosis through the activation of Nrf2 signaling pathways (Li et al., 2018[40]). Recently, we found that Pacific oyster (*C. gigas*) extracts fermented with *Lactobacillus brevis* BJ20 (referred to as fermented oyster extracts, FO) had better protection against liver damage than before fermentation (unpublished data). In our previous studies for the beneficial use of FO, we reported that FO strongly prevented RANKL-induced osteoclast differentiation through the inhibition of ROS generation (Jeong et al., 2019[29]). FO also effectively attenuated the induction of the osteoclast-specific genes required for osteoclastogenesis and bone resorption (Ihn et al., 2019[27]). Furthermore, it has been reported that FO activated the Wnt/β-catenin signaling pathway to promote osteoblast differentiation, leading to bone formation (Molagoda et al., 2019[44]). However, to date, whether FO can weaken oxidative stress-induced injury in osteoblasts has not been studied. Therefore, in this study, we evaluated the antioxidant potential of FO against oxidative stress (H_2_O_2_)-induced DNA damage and apoptosis in MC3T3-E1 osteoblasts. In addition, we explored the relevance of the Nrf2 signaling pathway to clarify the potential mechanism of FO-mediated antioxidant protection capacity. 

## Materials and Methods

### Reagents and antibodies 

Alpha modification of Eagle's minimum essential medium (α-MEM), fetal bovine serum (FBS), antibiotics mixtures, and other cell culture reagents were obtained from WelGENE Inc. (Gyeongsan, Republic of Korea). H_2_O_2_, 3-(4,5-dimethylthiazol-2-yl)-2,5-diphenyltetra-zolium bromide (MTT), 5,5',6,6'-tetrachloro-1,1'3,3'-tetraethyl-imidacarbocyanune iodide (JC-1), 4,6-diamidino-2-phenylindole (DAPI) and N-acetyl-L-cysteine (NAC) were purchased from Sigma-Aldrich Chemical Co. (St. Louis, MO, USA). Lactate dehydrogenase (LDH) cytotoxicity assay kit was supplied by Cayman Chemical Co. (Ann Arbor, MI, USA). Annexin V-fluorescein isothiocyanate (FITC) apoptosis detection kit and 2',7'-dichlorofluorescein diacetate (DCFDA) were purchased from R&D Systems Inc. (Minneapolis, MN, USA) and Molecular Probes (Eugene, OR, USA), respectively. Bradford assay reagent and the mitochondrial fractionation kit were obtained from Bio-Rad Laboratories (Hercules, CA, USA) and Active Motif, Inc. (Carlsbad, CA, USA), respectively. Polyvinylidene difluoride (PVDF) membranes were purchased from Merck Millipore (Bedford, MA, USA). Nrf2 siRNA and negative-control siRNA were purchased from Santa Cruz Biotechnology, Inc. (Santa Cruz, CA, USA). Lipofectamine 2000 reagent was obtained from Invitrogen (Carlsbad, CA, USA). HT 8-oxo-dG ELISA Kit II and Genomic DNA purification kit were supplied by Trevigen (Gaithersburg, MD, USA) and Promega Corporation (Madison, WI, USA), respectively. The *in vitro* caspase colorimetric assay kits and enhanced chemiluminescence (ECL) detection system were obtained from R&D Systems and Amersham Life Science (Arlington Heights, IL, USA), respectively. Primary antibodies were purchased from Abcam, Inc. (Cambridge, MA, UK), Cell Signaling Technology (Danvers, MA, USA), and Santa Cruz Biotechnology, Inc. Horseradish peroxidase (HRP)-conjugated secondary antibodies were obtained from Amersham Life Science. All other chemicals not specifically cited here were supplied by Sigma-Aldrich Chemical Co. Commercial FO (product name: FO100), extracted according to a modified version of the method used by Choi et al. (2016[9]), was kindly supplied by the Marine Bioprocess Co. (Busan, Republic of Korea). Before use in the experiments, FO was diluted in α-MEM to adjust final treatment concentration.

### Cell culture and cell viability assay

Mouse osteogenic MC3T3-E1 cells were obtained from the American Type Culture Collection (Manassas, VA, USA), and cultured in α-MEM supplemented with 10 % heat-inactivated FBS and antibiotics mixture in a water-saturated humidified incubator at 5 % CO_2_ and 37 °C. For measurement of cell viability, MC3T3-E1 cells were treated with various concentrations of FO for 24 h, or pretreated with different concentrations of FO or NAC for 1 h, and then incubated with or without 300 μM H_2_O_2_ for 24 h. The MTT solution was then added to a final concentration of 0.5 mg/ml at 37 °C for 3 h. At the end of the incubation, the culture supernatants were carefully discarded from the culture plate, and the formazan crystals formed were dissolved in dimethyl sulfoxide (DMSO). Optical density was measured at 570 nm absorbance using an enzyme-linked immunosorbent assay (ELISA) reader (Dynatech Laboratories, Chantilly, VA, USA). The optical density of the formazan crystals formed in the untreated control cells was used to represent 100 % viability (Kim et al., 2019[33]). 

### Nuclear staining

To determine apoptosis, the changes of nuclear morphology were examined using DAPI staining. Briefly, cells were harvested after treatment with H_2_O_2_ in the absence or presence of FO or NAC, washed with phosphate-buffered saline (PBS), and then fixed with 3.7 % paraformaldehyde in PBS for 10 min at room temperature (RT). The cells were washed with PBS again, and stained with 2.5 µg/ml DAPI solution for 10 min at RT. The cells were observed *via* fluorescence microscopy (Carl Zeiss, Oberkochen, Germany).

### Detection of apoptosis by annexin V staining

The magnitude of the apoptosis was detected using the annexin-V FITC apoptosis detection kit, according to the manufacturer's instructions. In brief, after treatment with H_2_O_2_ in the absence or presence of FO or NAC, the collected cells were washed with cold PBS, fixed in 75 % ethanol at 4 °C for 30 min, and then stained with annexin V-FITC and propidium iodide (PI) for 20 min at RT in the dark. Using a flow cytometer (Becton Dickinson, San Jose, CA, USA), the fluorescence intensities of the cells were quantified as the percentage of annexin V-positive and PI-negative (Annexin V^+^/PI^-^) cell populations, as indicators of apoptotic cells, while the V^-^/PI^-^ cell population was considered as normal (Kim et al., 2019[32]) . 

### LDH release assay

The content of LDH in the culture medium, which is a well-known indicator of cell membrane injury and cell cytotoxicity, was assayed using a commercial kit, based on the manufacturer's instructions. Briefly, 100 μL of culture supernatant was collected from each well, transferred to new plates, and then 100 μL of LDH reaction solution buffer supplied in the kit was added. After gentle mixing at RT for 30 min, absorbance was measured at 490 nm for each sample using an ELISA plate reader, and calculated according to the formula included in the kit.

### Western blot analysis 

Cells were collected and lysed on ice for 30 min in ice-cold lysis buffer [5 mM Na-ethylenediaminetetraacetic acid (Na-EDTA), 25 mM Tris-Cl (pH 7.5), 250 mM NaCl, 1 mM phenylmethylsulfonyl fluoride, 1 % nonidet-P40, and 5 mM dithiothreitol]. In a parallel experiment, the mitochondrial and cytosolic proteins were isolated using a mitochondrial fractionation kit, according to the manufacturer's procedure. Protein concentration of the collected supernatants was measured using the Bradford assay reagent according to the manufacturer's protocol. Subsequently, the same amount of protein from each sample was separated by sodium-dodecyl sulfate (SDS)-polyacrylamide gel electrophoresis, and transferred to PVDF membranes. The membranes were blocked with Tris-buffered saline (10 mM Tris-Cl, pH 7.4) containing 5 % skim milk and 0.5 % Tween-20 for 1 h at RT, and then incubated overnight at 4 °C with primary antibodies. After washing with PBS, the membranes were incubated with the appropriate HRP-conjugated secondary antibodies for 2 h at RT. Protein bands were detected using an ECL detection system and chemiluminescence imaging (Azure Biosystems, Inc., Dublin, CA, USA).

### Small interfering RNA (siRNA) transient transfection

siRNA transient transfection for Nrf2 gene inhibition was performed using Lipofectamine 2000 reagent, according to the manufacturer's instructions. Briefly, cells were seeded at a density of 3 × 10^4^ cells per well in 6-well plates with fresh cell culture medium. The cells were transfected with targeted Nrf2 siRNA, and control siRNA as a negative control, using Lipofectamine 2000 reagent in serum-free medium for 6 h. Following transfection, the reagent was replaced with normal growth medium, and the cells were incubated for an additional 24 h, and then treated with H_2_O_2_ in the absence or presence of FO or NAC for the indicated periods.

### Measurement of ROS generation 

To measure the amount of ROS generated in cells, cells were seeded onto 6-well plates with density of 3×10^5^ cells per well, and treated with or without FO and NAC for 1 h, before another 1 h culture with the addition of H_2_O_2_. The cells were washed with PBS, and lysed with PBS containing 1 % Triton X-100 for 10 min at 37 °C. They were then stained with 10 µM DCFDA for 30 min at RT in the dark, and washed with PBS. Intracellular ROS generation was immediately recorded at 515 nm by a flow cytometer. The results were expressed as the percentage increase relative to untreated cells. 

### Comet assay

A comet assay, a single-cell gel electrophoresis assay for DNA damage, was used to analyze DNA migration from individual cells in the gel. After cells were exposed to H_2_O_2_ with or without FO, the cells were suspended in 0.5 % low melting point agarose (LMA) at 37 °C, aliquoted, and then spread onto fully frosted glass microscope slides precoated with 1 % normal melting agarose. After the agarose are solidified in the dark, the slide was covered with 0.5 % LMA, and submerged in lysis solution (2.5 M of NaCl, 100 mM of Na-EDTA, 1 % Trion X-100, 10 mM of Tris, and 10 % DMSO, pH 10) for 1 h at 4 °C. The slides were then incubated for 30 min in a gel electrophoresis device containing 300 mM NaOH and 10 mM Na-EDTA (pH 13), and electrophoretically examined at 30 V (1 V/cm) and 300 m Amp for 20 min, to draw negatively charged DNA toward the anode. Slides were subsequently washed three times with neutralizing buffer, 0.4 M of Tris (pH 7.5) at 4 °C, and stained with 20 µg/ml of PI. The slides were observed under fluorescence microscopy, and the resulting images were analyzed.

### Determination of 8-hydroxy-2'-deoxyguanosine (8-OHdG) concentration

The levels of intracellular 8-OHdG, a ubiquitous marker of DNA damage by oxidative stress, were quantitated using an HT 8-oxodG ELISA Kit II. In brief, total genomic DNA was isolated from cells using the Genomic DNA purification kit, according to the manufacturer's instructions. Extracted DNA was quantitated, and adjusted to the final concentration at 200 µg/ml in each sample. Then, DNA was digested by DNase I and alkaline phosphatase sequentially for 1 h at 37 °C. For the determination of 8-OHdG in culture supernatants, cell culture medium was clarified for use after centrifugation of cell debris, and the amount of 8-OHdG was measured using an ELISA plate reader at 450 nm, based on the manufacturer's instructions. Subsequently, the concentration of 8-OHdG for each sample was quantified from the standard curve.

### Measurement of mitochondrial membrane potential (MMP, Δψ_m_)

The mitochondrial function was identified by membrane potential fluorescence staining using the mitochondrial potential sensor JC-1. Briefly, after treatment with H_2_O_2_ in the absence or presence of FO, the cells were trypsinized. The collected pellets were suspended in 500 µL of PBS, and incubated with 10 µM JC-1 at 37 °C for 20 min. The cells were then washed with cold PBS, and analyzed using a flow cytometer.

### Determination of caspase-3 and caspase-9 enzymatic activities

The activities of caspase-3 and caspase-9 were determined by using colorimetric activity assay kits, according to the manufacturer's instructions. Briefly, the cells were incubated in a supplied lysis buffer on ice for 15 min, and then the lysates were centrifuged at 12,000×*g* for 15 min. The supernatants were collected, and a total of 200 μg protein lysate was incubated with 5 μL of caspase-3 [Acetyl (Ac)-Asp (D)-Glu (E)-Val (V)-Asp (D)-*p*-nitroanilide (*p*NA)] or caspase-9 [Ac- Leu (L)-Glu (E)-His (H)-Asp (D)-pNA] substrate in the dark for 2 h at 37 °C, according to the kit protocol. The concentrations of *p*NA released from the substrate by caspase-3 and caspase-9 were calculated from the absorbance values at 405 nm. According to their concentration curve, the results of at least three independent experiments were expressed as fold change, compared with the untreated control cells.

### Statistical analysis 

The results were expressed as the mean ± standard deviation (SD) of at least three independent experiments. Statistical analyses were performed using the SPSS software, version 16.0 (SPSS Inc., Chicago, IL, USA). The statistical significance was analyzed by one-way ANOVA. A value of *p* < 0.05 was considered to indicate a statistically significant difference. 

## Results

### FO inhibited H_2_O_2_-induced cytotoxicity in MC3T3-E1 cells

**Figure 1:**
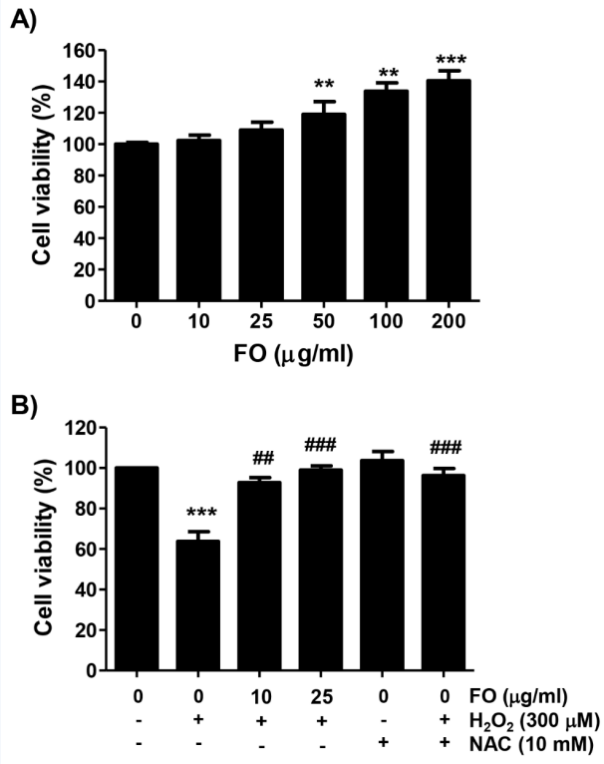
Protective effect of FO on the H_2_O_2_-induced cytotoxicity in MC3T3-E1 cells. Cells were treated with the various concentrations of FO for 24 h (A), or pretreated with or without FO or NAC at the indicated concentrations for 1 h, and then cultured in the presence of 300 μM H_2_O_2_ for 24 h (B). The cell viability was determined by MTT reduction assay. The results were expressed as the mean ± SD obtained from three independent experiments (** *p *< 0.01 and *** *p *< 0.001 compared with the control group, ## *p *< 0.01 and ### *p *< 0.001 compared with the H_2_O_2_-treated group).

A series of studies demonstrated that H_2_O_2_ could promote DNA damage and apoptosis, as well as bone loss, by inducing the self-generation of free radicals in osteoblasts (Li et al., 2019[42]; Yao et al., 2018[62]; Fu et al., 2015[16]), so H_2_O_2_ was used to induce oxidative stress in MC3T3-E1 cells. To examine the effects of FO on the proliferation of MC3T3-E1 cells, the cells were stimulated with various concentrations of FO for 24 h, and the cell viability was then determined by MTT assay. Figure 1A(Fig. 1) shows that in the cells treated with FO below 25 µg/ml, there was no significant difference in cell proliferation compared to the control, but FO over the concentration of 50 µg/ml significantly increased the proliferation of MC3T3-E1 cells, which is consistent with our previous study (Molagoda et al., 2019[44]). Therefore, to study the cytoprotective effects of FO against H_2_O_2_-induced oxidative stress, a concentration of FO of less than 25 µg/ml with no significant change in cell proliferation was chosen. On the other hand, the viability of MC3T3-E1 cells exposed to H_2_O_2_ was significantly reduced in a concentration-dependent manner (data not shown), and the concentration of H_2_O_2_ for the induction of cytotoxicity was selected as 300 μM, with a survival rate of about 60 % (Figure 1B(Fig. 1)). We then examined the ability of FO to counteract H_2_O_2_-induced oxidative stress, and found that FO inhibited the H_2_O_2_-mediated loss of MC3T3-E1 cell viability in a concentration-dependent manner. We also found that pretreatment of NAC, a well-established ROS scavenger, had a complete inhibitory effect on H_2_O_2_-induced cytotoxicity, when compared to the control (Figure 1B(Fig. 1)).

### FO attenuated H_2_O_2_-induced apoptosis in MC3T3-E1 cells

**Figure 2:**
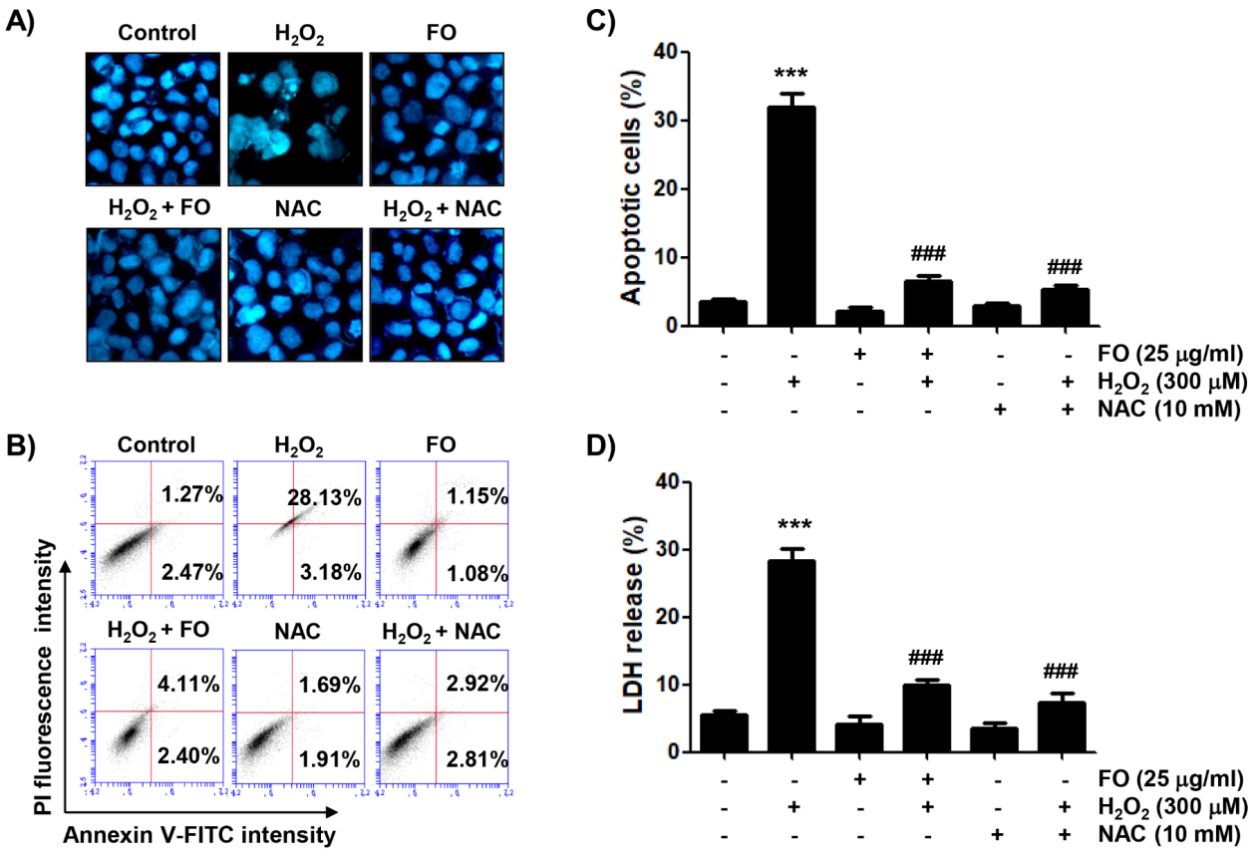
Inhibitory effect of FO on H_2_O_2_-induced apoptosis in MC3T3-E1 cells. Cells were pretreated with or without 25 μg FO or 10 mM NAC for 1 h, before treatment with 300 μM H_2_O_2_ for 24 h. (A) The cells were stained with DAPI solution, and the stained nuclei were observed by fluorescence microscopy (original magnification, ×200). Each image is representative of at least three independent experiments. (B) and (C) The cells were fixed and stained with annexin V-FITC and PI for flow cytometry analysis. (B) The results show early apoptosis, defined as annexin V^+^ and PI^-^ cells (lower right quadrant), and late apoptosis, defined as annexin V^+^ and PI^+^ (upper right quadrant) cells, and representative profiles are shown. (C) The percentages of apoptotic cells were determined by expressing the numbers of annexin V^+^ cells as percentages of all the present cells. (D) LDH release into the extracellular medium was measured to determine cytotoxicity. Data represent the mean ± SD of three independent experiments (*** *p *< 0.001 compared with the control group, ### *p *< 0.001 compared with the H_2_O_2_-treated group).

Because the inhibition of MC3T3-E1 cell proliferation by H_2_O_2_ treatment is closely associated with the induction of apoptosis (Li et al., 2019[42]; Yao et al., 2018[62]), we investigated the ability of FO to prevent H_2_O_2_-induced apoptosis using DAPI staining and annexin V-FITC/PI double staining assays. Figure 2A(Fig. 2) shows that apoptotic nuclei, characterized by bright blue chromatin that was highly condensed or fragmented, by DAPI staining were clearly observed in H_2_O_2_-treated cells. However, pretreatment with FO or NAC in H_2_O_2_-treated cells markedly reduced these morphological changes. In addition, the results of flow cytometric analysis showed that H_2_O_2_ triggered a higher magnitude of apoptosis, compared to the control (Figures 2B and C(Fig. 2)). However, after pretreatment with NAC as well as FO, the percentage of apoptotic cells significantly dropped. Furthermore, in the LDH assay, cells exposed to H_2_O_2_ showed a marked increase in LDH release, while there was a decreasing trend in FO-pretreated cells, as for NAC-pretreated cells (Figure 2D(Fig. 2)), indicating that FO strongly protected against MC3T3-E1 cell apoptosis by H_2_O_2_-induced oxidative stress.

### FO activated the Nrf2/HO-1 signaling pathway and alleviated H_2_O_2_-induced ROS generation in MC3T3-E1 cells

**Figure 3:**
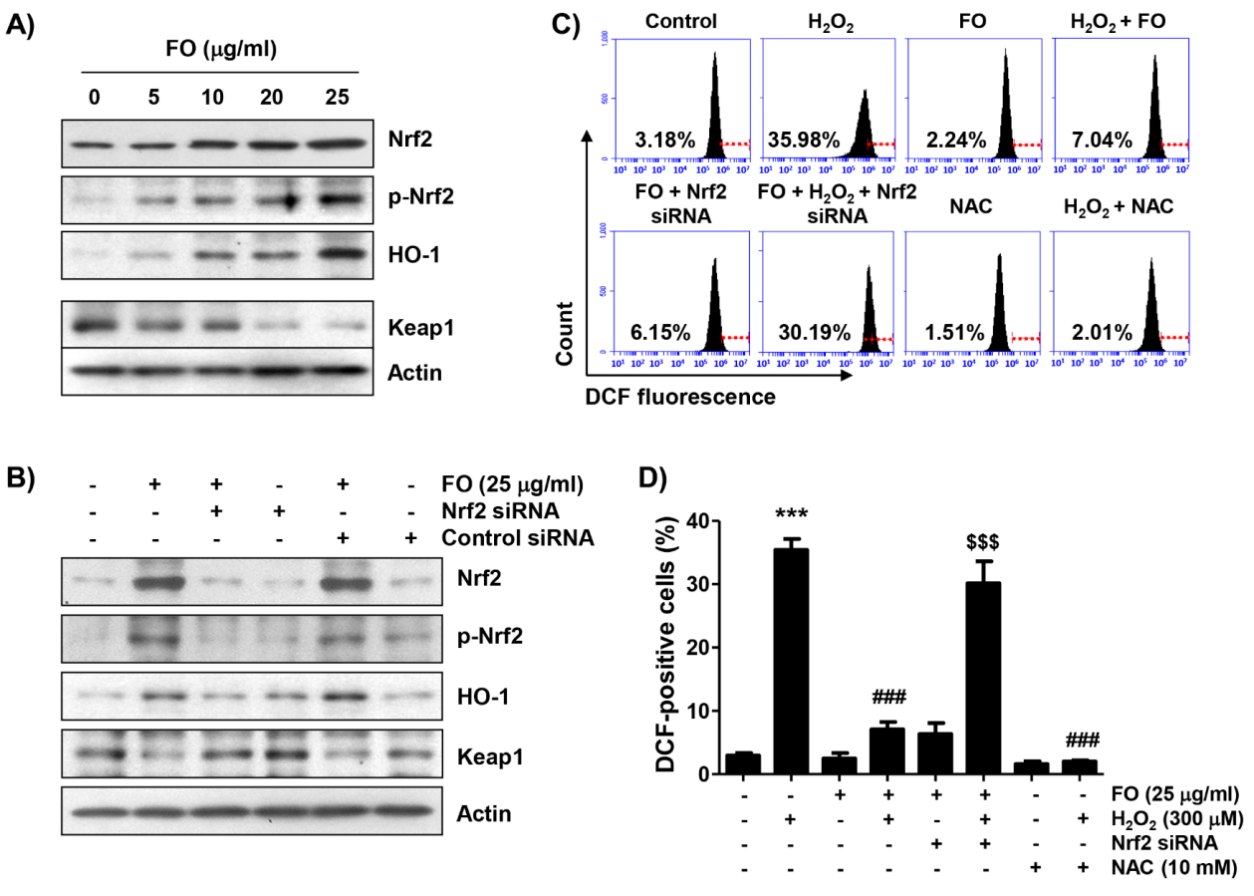
Activation of Nrf2 signaling pathway and inhibition of H_2_O_2_-induced ROS generation by FO in MC3T3-E1 cells. (A) Cells were stimulated with the indicated concentrations of FO for 24 h. (B) Cells transfected with either negative control siRNA or Nrf2 siRNA were treated with 25 μg/ml FO for 24 h. (A) and (B) Equal amounts of proteins were separated by SDS-polyacrylamide gel electrophoresis, and subjected to Western blot analysis of the listed proteins. Actin was used as an internal control, and the proteins were visualized using an ECL detection system. (C) and (D) After transfection with or without Nrf2-siRNA, the cells were pretreated with 25 μg/ml FO or 10 mM NAC for 1 h, and then treated with or without 300 μM H_2_O_2_ for 1 h. The medium was removed, and the cells were incubated for 20 min with medium containing 10 µM DCFDA for 20 min. (C) ROS production was measured by flow cytometry, and representative profile is shown. The red line is indicating the cut-off for control cells. (D) The measurements were made in triplicate, and the values are expressed as the mean ± SD (*** *p *< 0.001 compared with the control group, ### *p *< 0.001 compared with the H_2_O_2_-treated group, $$$ *p *< 0.001 compared with the FO and H_2_O_2_-treated group).

Previous reports have suggested that the activation of the Nrf2/HO-1 signaling is an important cytoprotective pathway against oxidative injury in osteoblasts (Xu et al., 2020[60]; Tian et al., 2019[53]; Kim et al., 2014[34]). Therefore, we examined whether the expression of Nrf2 and HO-1 proteins is increased in FO-treated MC3T3-E1 cells. According to the results of Western blot analysis, the expression of Nrf2 and its phosphorylation level (p-Nrf2) continued to increase with increasing FO treatment concentration, compared with the untreated control group (Figure 3A(Fig. 3)). At the same time, the expression of HO-1 protein also gradually increased; but Keap1 expression, which binds to Nrf2 as a negative regulator of Nrf2 (Shaw and Chattopadhyay, 2020[51]; Fetoni et al., 2019[15]), was relatively decreased, indicating that FO treatment activated the Nrf2/HO-1 signaling pathway. To clarify whether the increased expression of HO-1 by FO is Nrf2-dependent, Nrf2 expression was blocked by Nrf2 siRNA transfection. Figure 3B(Fig. 3) indicates that the knockdown of Nrf2 efficiently decreased the expression of Nrf2, as well as HO-1 promoted by FO, while Nrf2 siRNA reversed the FO-mediated reduction of Keap-1 expression. As is well known, because the cytotoxicity of H_2_O_2_ in osteoblasts is associated with the overproduction of ROS (Li et al., 2019[42]; Fu et al., 2015[16]), we next investigated whether FO inhibited H_2_O_2_-induced ROS accumulation using a fluorescent probe, DCFDA, and found that the production of ROS in H_2_O_2_-treated cells peaked within 1 h (about 11-fold more than the control, Figure 3C(Fig. 3)), and gradually decreased with increasing time (data not shown). However, pretreatment with FO significantly alleviated the effect of H_2_O_2_ on ROS overproduction, and NAC also almost completely inhibited the accumulation of ROS (Figures 3C and D(Fig. 3)). Furthermore, depletion of Nrf2 reversed the blocking of intracellular ROS production by FO in H_2_O_2_-treated cells, suggesting that FO exerts antioxidant properties by activating Nrf2.

### Nrf2 is involved in the reduction of H_2_O_2_-induced DNA damage by FO in MC3T3-E1 cells

**Figure 4:**
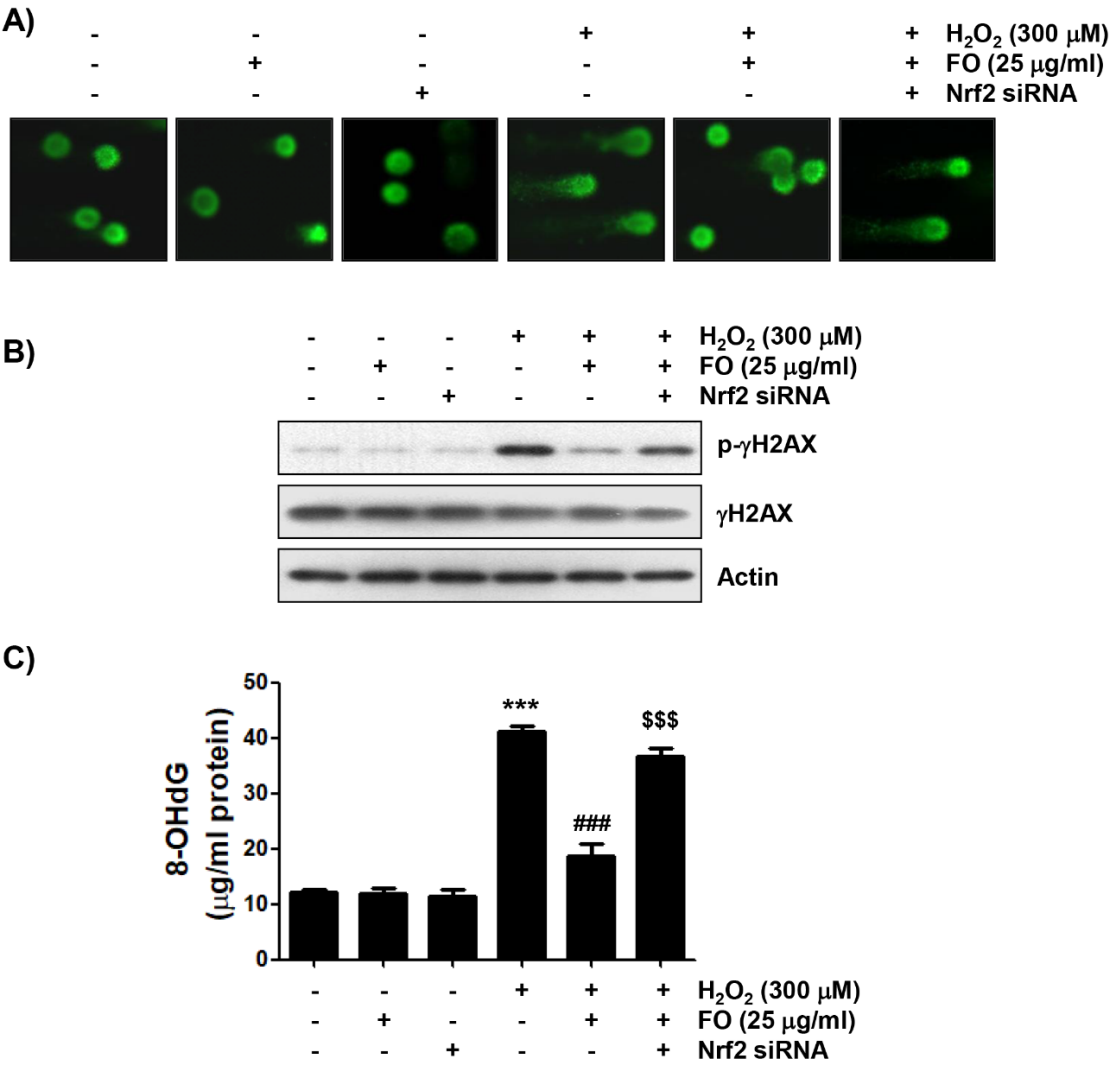
Protection of H_2_O_2_-induced DNA damage by FO in MC3T3-E1 cells. Cells transfected with or without Nrf2-siRNA were pretreated with 25 μg/ml FO for 1 h, and then treated with or without 300 μM H_2_O_2_ for 24 h. (A) Comet assay was performed, and representative images were captured by fluorescence microscopy (original magnification, ×400). (B) Total cell lysates were prepared, and p-γH2AX and γH2AX expressions were identified by Western blot analysis. The equivalent loading of proteins in each well was confirmed by actin. (C) The DNA samples of cells were subjected to assessment of 8-OHdG level. The measurements were made in triplicate, and the results are expressed by mean ± SD (*** *p *< 0.001 compared with the control group, ### *p *< 0.001 compared with the H_2_O_2_-treated group, $$$ *p *< 0.001 compared with the FO and H_2_O_2_-treated group).

Since cellular DNA damage due to free radical production contributes to the induction of apoptosis, we investigated whether FO can block DNA damage caused by H_2_O_2_. We first conducted the alkaline comet assay, a sensitive method to assess DNA strand breaks in single cells (Kuchařová et al., 2019[39]; Fairbairn et al., 1995[14]), in MC3T3-E1 cells exposed to H_2_O_2_, following pretreatment with FO. As shown in the representative images indicated in Figure 4A(Fig. 4), there was no apparent comet tail moment (DNA migration) in cells treated with FO alone or control cells, and markedly increased DNA migration in H_2_O_2_-treated cells. However, while the DNA migration by H_2_O_2 _was clearly reduced to the control levels in the FO pretreatment cells, the ability of FO to inhibit DNA migration was offset in cells that artificially inhibited Nrf2 expression. Furthermore, the DNA damage blocking effect of FO was reconfirmed by the analysis of the phosphorylation of γH2AX (Ser139, p-γH2AX), a sensitive marker for DNA double strand breaks (Kopp et al., 2019[38]; Georgoulis et al., 2017[19]), and 8-OHdG production, an oxidative DNA damage marker (Dąbrowska and Wiczkowski, 2017[10]; Di Minno et al., 2016[11]). Figures 4B and C(Fig. 4) show that although there was no significant change in the total protein expression of γH2AX in cells treated with H_2_O_2 _alone, the expression of p-γH2AX was obviously increased, and higher levels of 8-OHdG were also found in the H_2_O_2 _treated cells, compared to the control. However, pretreatment of FO clearly attenuated the H_2_O_2_-induced phosphorylation of γH2AX, and significantly suppressed the production of 8-OHdG. On the other hand, Nrf2 knockdown antagonized the reduced expression of p-γH2AX and content of 8-OHdG by FO in H_2_O_2_-treated cells. These results confirm that the activation of Nrf2/HO-1 signal pathway is involved in the protective effect of FO against H_2_O_2_-induced DNA damage in MC3T3-E1 cells.

### The protective effect of H_2_O_2_-induced mitochondrial dysfunction by FO is related to the activation of Nrf2 signaling pathway

**Figure 5:**
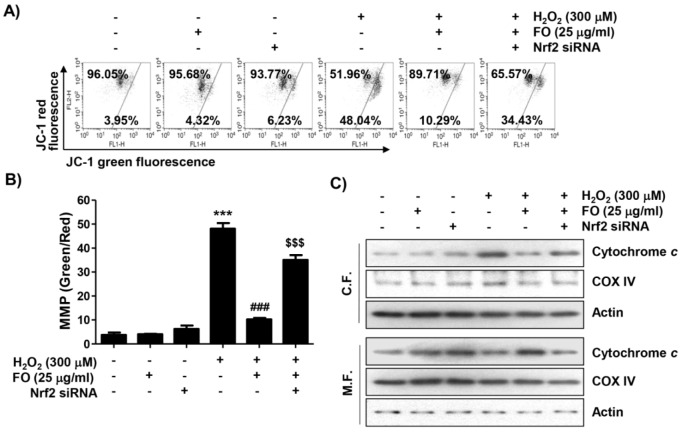
Attenuation of H_2_O_2_-induced mitochondrial dysfunction by FO in MC3T3-E1 cells. Cells transfected with or without Nrf2-siRNA were treated with 25 μg/ml FO for 1 h, then exposed to 300 μM H_2_O_2_ for 24 h. (A) The cells were collected and stained with 10 μM JC-1 for 20 min. JC-1 fluorescence intensity was detected for evaluation of the changes of MMP using flow cytometry. (B) The green (JC-1 monomers) and red (JC-1 aggregates) fluorescence ratio is indicating the proportion of mitochondrial depolarization. The data represent the mean ± SD of triplicate determinations (*** *p *< 0.001 compared with the control group, ### *p *< 0.001 compared with the H_2_O_2_-treated group, $$$ *p *< 0.001 compared with the FO and H_2_O_2_-treated group). (C) The expression of cytochrome *c* was analyzed by Western blot analysis using mitochondria and cytoplasmic fractions isolated from cells cultured under the same conditions. Actin and cytochrome oxidase subunit VI (COX IV) serve as protein loading controls for the cytosol and mitochondria, respectively.

Excessive ROS is well known as a hallmark of mitochondrial damage. Also, loss of mitochondrial permeability regulated by MMP contributes to the onset of activation of the intrinsic apoptosis pathway induced by oxidative stress (Indo et al., 2017[28]; Xiong et al., 2014[59]). Therefore, to analyze whether the inhibition of mitochondrial impairment is a mechanism involved in the cytoprotective effect of FO, JC-1 was used to estimate the MMP. As indicated in Figures 5A and B(Fig. 5), a significant decrease in the ratio of JC-1 green fluorescence signal with a decrease in JC-1 red fluorescence signal was observed in MC3T3-E1 cells exposed to H_2_O_2_, indicating that the depolarization of MMP was induced. Meanwhile, at the initiation of the intrinsic apoptosis pathway caused by mitochondrial dysfunction due to the loss of MMP, the cytoplasmic release of cytochrome *c* from mitochondria is increased (Bock and Tait, 2020[3]; Xiong et al., 2014[59]). As expected, the expression of cytochrome* c* in H_2_O_2_-treated cells increased in the cytoplasmic fraction, and decreased in the mitochondrial fraction (Figure 5C(Fig. 5)), indicating that cytochrome *c* was released from the mitochondria to the cytosol. Also, pretreatment of FO significantly reversed H_2_O_2_-induced loss of MMP, but the protective effect of FO was abolished in Nrf2 siRNA-transfected cells. Concomitant with these results, the inhibitory effect of H_2_O_2_-induced cytosolic release of cytochrome *c* by FO also disappeared with Nrf2 knockdown (Figure 5(Fig. 5)). These results indicate that the protective effect of FO against H_2_O_2_-induced mitochondrial dysfunction in MC3T3-E1 cells was mediated through Nrf2-dependent pathway.

### Nrf2 was involved in the mitigation of H_2_O_2_-mediated apoptosis by FO in MC3T3-E1 cells

**Figure 6:**
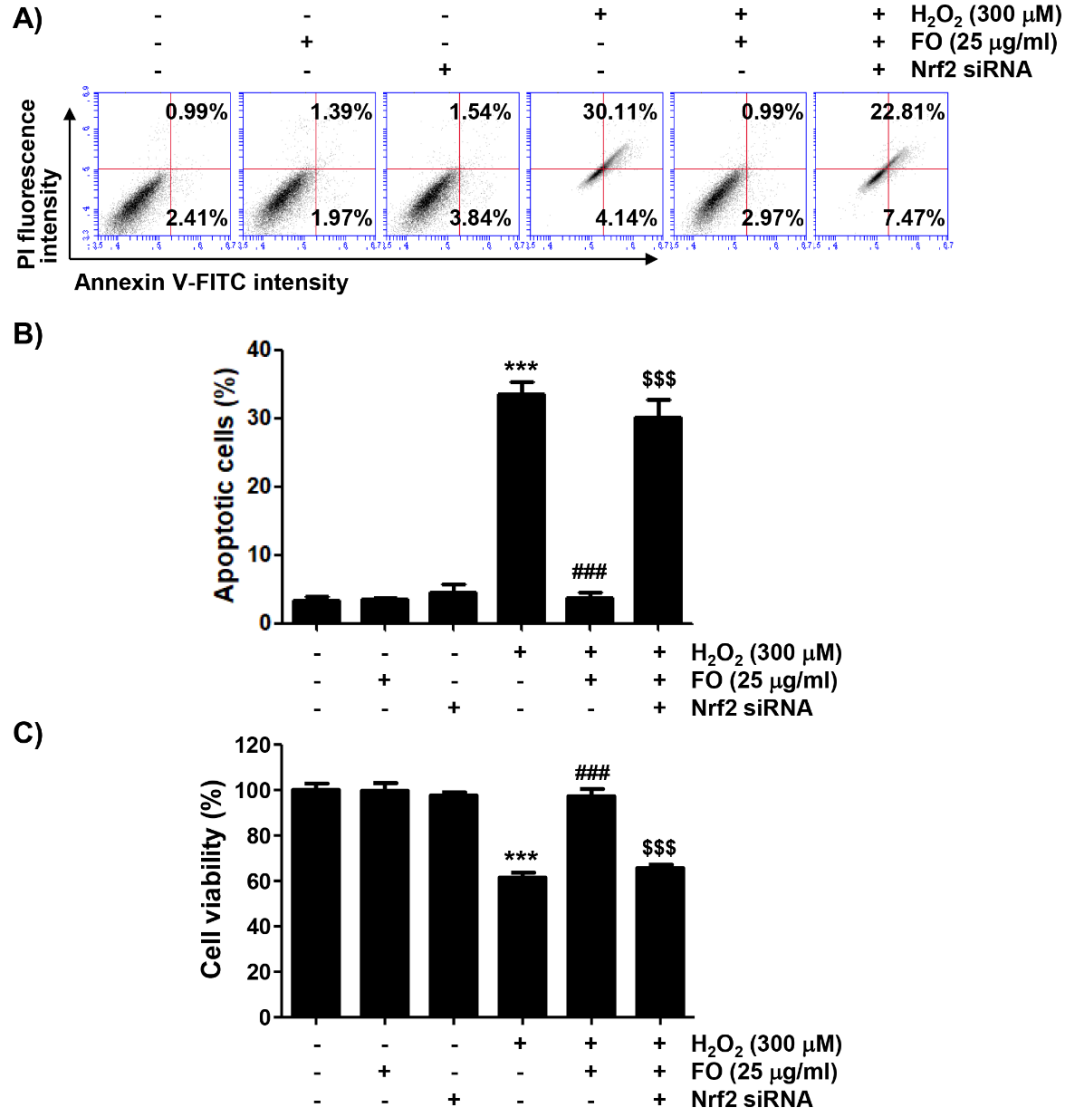
Prevention of H_2_O_2_-induced apoptosis by FO was dependent on the activation of Nrf2 signaling pathway in MC3T3-E1 cells. Cells transfected with or without siRNA-Nrf2 were treated with 25 μg/ml FO for 1 h, then exposed to 300 μM H_2_O_2_ for 24 h. (A) The cells were stained with annexin V-FITC and PI for flow cytometry analysis, and representative profiles are presented. (B) The percentages of apoptotic cells were determined by counting the percentages of annexin V^+^ cells. (C) The cell viability was determined by MTT assay. The results are expressed as the mean ± SD of three independent experiments (*** *p *< 0.001 compared with the control group, ### *p *< 0.001 compared with the H_2_O_2_-treated group, $$$ *p *< 0.001 compared with the FO and H_2_O_2_-treated group).

**Figure 7:**
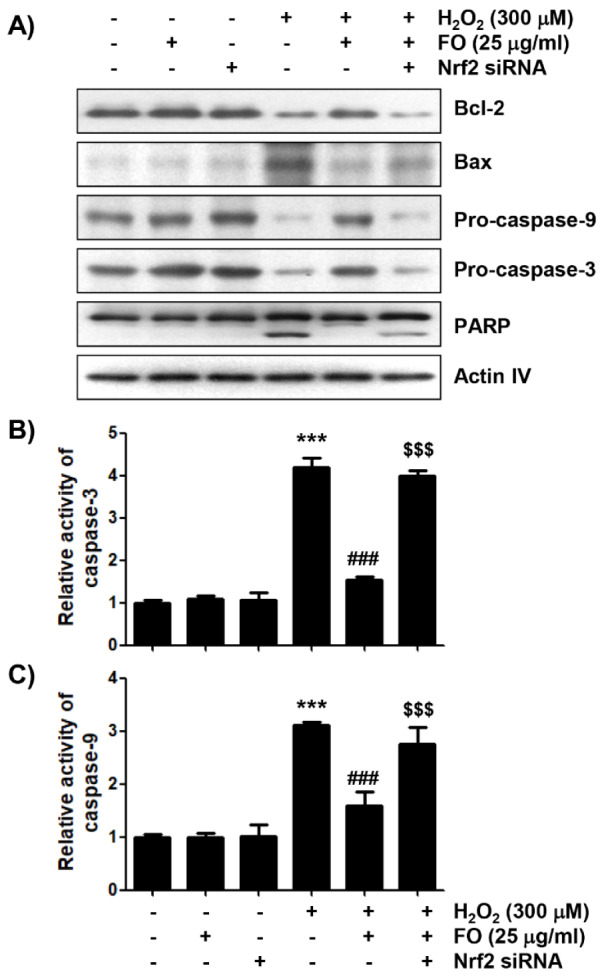
The effects of FO on the expression levels of apoptosis-related proteins, and the activity of caspases in H_2_O_2_-treated MC3T3-E1 cells. (A) The cells cultured under the same conditions as Figure 6 were lysed, and Western blot analysis was performed using the indicated antibodies. Actin was used as an internal control. (B) and (C) The activities of caspase-3 and caspase-9 in cell lysates were measured using the respective substrate peptides, Ac-DEVD-*p*NA and Ac-LEHD-pNA. Data are expressed as the mean ± SD obtained from three independent experiments (*** *p *< 0.001 compared with the control group, ### *p *< 0.001 compared with the H_2_O_2_-treated group, $$$ *p *< 0.001 compared with the FO and H_2_O_2_-treated group).

Finally, we investigated whether the Nrf2/HO-1 signaling pathway was involved in the anti-apoptotic effect of FO in MC3T3-E1 cells. Figures 6A and B(Fig. 6) show that the anti-apoptotic effect of FO was significantly lost by treatment with Nrf2 siRNA in H_2_O_2_-insulted cells. At the same time, Nrf2 siRNA transfection eventually eliminated the protective effect of FO against the H_2_O_2_-induced reduction of cell viability (Figure 6C(Fig. 6)). Cytochrome *c* release into the cytoplasm is evident in the initial activation of the intrinsic apoptotic pathway, thereby completing apoptosis through the degradation of various intracellular substrate proteins, following activation of the caspase cascade. This process is also controlled by Bcl-2 family proteins involved in promoting or inhibiting apoptosis (Bock and Tait, 2020[3]; Popgeorgiev et al., 2018[47]; Er et al., 2006[13]). As indicated in Figure 7A(Fig. 7), the results of immunoblot analysis revealed that the expression of anti-apoptotic Bcl-2 protein was decreased, and that of pro-apoptotic Bax was increased in H_2_O_2_-treated cells, but their expression in cells pretreated with FO was maintained at control levels. In addition, as the expression of pro-caspase-9 and pro-caspase-3 markedly decreased in H_2_O_2_-treated cells, their enzymatic activity increased significantly; and degradation of poly (ADP-ribose) polymerase (PARP), a representative substrate protein degraded by activated effector caspases (Kiraz et al., 2016[36]; Hassan et al., 2014[23]), also increased. Moreover, these effects were significantly restored in cells cultured in medium containing FO. However, in the condition that Nrf2 expression was blocked, the expression rate of Bax to Bcl-2 was increased again, and the activity of caspases was significantly maintained, resulting in an increase in the degradation of PARP (Figure 7(Fig. 7)). These results suggest that the activation of the Nrf2/HO-1 signaling pathway may act as an upstream signal on the protective potential of FO against oxidative stress-induced MC3T3-E1 cell apoptosis.

## Discussion

Oxidative stress, characterized by the overproduction of ROS in cells or tissues, is closely related to the reduction of bone mineral density, as well as the occurrence of bone loss by damage to osteoblasts and osteocytes (Schröder, 2019[50]; An et al., 2016[2]). Mitochondria are most vulnerable to excessive H_2_O_2_ insults among intracellular organelles; and their dysfunction contributes significantly to ROS production, causing oxidative damage to cellular components, including proteins, lipids, and nucleic acids. Excessive ROS also leads to the formation of protein-protein crosslinks, and the oxidation of amino acid residues that cause various pathological phenomena, including osteoporosis (Indo et al., 2017[28]; Xiong et al., 2014[59]). Therefore, the search for novel antioxidants to inhibit oxidative damage-mediated osteoblastic disorders has emerged as a strategy for the prevention and treatment of osteoporosis. In the present study, we exposed H_2_O_2_ to induce oxidative damage in MC3T3-E1 cells, and found that H_2_O_2_ induced cytotoxicity by triggering DNA damage and mitochondria-mediated apoptosis through the promotion of ROS production. However, FO was found to have the ability to revise H_2_O_2_-induced DNA damage and apoptosis, while having ROS scavenging activity.

Recently, the link between Nrf2 deficiency and osteoporosis is supported by increasing evidence. For example, loss of Nrf2 increases the susceptibility of bone loss due to increased oxidative stress (Liu et al., 2019[43]; Pellegrini et al., 2017[46]; Ibáñez et al., 2014[26]); and in contrast, loss of Nrf2 accelerates bone loss, by promoting RANKL-induced osteoclast differentiation or upregulating RANKL (Hyeon et al., 2013[25]; Rana et al., 2012[49]). In addition, various natural products have been reported to have antioxidant properties through the activation of Nrf2/HO-1 signaling pathway, to protect osteoblast apoptosis caused by oxidative injury (Jin et al., 2020[30]; Wang et al., 2019[55]; Cheng et al., 2019[8]; Han et al., 2019[22], 2017[20]). Therefore, the effect of FO on the expression of Nrf2 was explored, and the results of Western blot analysis showed that the expression of p-Nrf2 protein was markedly upregulated, meaning that Nrf2 was activated in FO-treated MC3T3-E1 cells. We used Nrf2 siRNA to further elucidate the role of Nrf2 in the antioxidant effects of FO, and found that FO increased HO-1 expression in a Nrf2-dependent manner, which was associated with a decrease in Keap1 expression. Concomitant with these results, when Nrf2 expression was blocked, ROS scavenging ability of FO was canceled, suggesting that the antioxidant potency of FO requires at least the activation of the Nrf2/HO-1 signaling pathway. In addition, although DNA damage accompanying H_2_O_2_-induced apoptosis was effectively weakened by FO, this protective effect disappeared under Nrf2 knockdown conditions. Therefore, our results clearly indicate that the increased HO-1 expression by FO contributed to the FO-mediated inhibition of H_2_O_2_-induced oxidative damage, supporting the findings of previous studies that the expression of HO-1 protein to counter oxidative stress in MC3T3-E1 cells is by Nrf2 activation (Han et al., 2019[22], 2018[21]; Xu et al., 2019[61]; Kook et al., 2015[37]; Kim et al., 2014[34]).

Mitochondria are important for osteoblast energy metabolism, and mitochondrial dysfunction due to oxidative stress is directly involved in the activation of the intrinsic apoptosis pathway (Indo et al., 2017[28]; Xiong et al., 2014[59]). Because osteoblasts are involved in the calcification of extracellular matrix, and are specialized in calcium transport, mitochondrial dysfunction of osteoblasts is also directly related to the induction of osteoporosis (Boonrungsiman et al., 2012[5]; Stambough et al., 1984[52]). ROS overload causes free radical attack of the phospholipid bilayer, which in turn leads to depolarization of the mitochondrial membrane, resulting in the loss of MMP as the mitochondrial membrane pores open. During this process, the permeability of mitochondrial membranes is increased, allowing apoptogenic factors, especially cytochrome *c*, in the mitochondrial intermembrane space to be released into the cytoplasm. Thus, loss of MMP and cytosolic release of cytochrome *c* are indicative of the impairment of mitochondrial function, and are phenomena that are evident early in the onset of mitochondria-mediated intrinsic apoptosis (Bock and Tait, 2020[3]; Er et al., 2006[13]). To evaluate the preventive effect of FO on mitochondrial dysfunction, MMP values ​​and cytochrome *c* expression were examined. The results of flow cytometry and Western blot analysis revealed that MMP was significantly decreased and cytosolic cytochrome *c* expression was markedly increased in H_2_O_2_-treated MC3T3-E1 cells. Also, while FO pretreatment prevented the reduction of MMP induced by H_2_O_2_ and maintained the expression of cytochrome *c* in mitochondria during H_2_O_2_ exposure, these protective effects were abolished in cells that blocked the expression of HO-1 by the knockdown of Nrf2. Consistent with these results, the reduced apoptosis and increased cell viability by pretreatment of FO in the presence of H_2_O_2_ were significantly reversed to the level of H_2_O_2_ alone-treated cells in Nrf2 siRNA-transfected cells. These results imply that protection of mitochondrial dysfunction and cytotoxicity by FO was achieved through the activation of Nrf2-dependent HO-1 signaling pathway in H_2_O_2_-treated MC3T3-E1 osteoblasts.

Apoptosis, a programmed cell death, is tightly regulated by a variety of factors, and can be divided into extrinsic and intrinsic pathways. The former is activated by extracellular ligands that bind to death receptors on the cell surface, while the latter is associated with the launch of intracellular apoptotic signals that cause mitochondrial dysfunction (Bock and Tait, 2020[3]; Popgeorgiev et al., 2018[47]). In particular, cytochrome *c*, released into the cytoplasm due to the loss of MMP, interacts with and activates caspase-9, which is a main initial step in the intrinsic apoptosis pathway. Activation of caspase-9 sequentially activates downstream effector caspases, such as caspase-3 and caspase-7, eventually completing cell death. This process is accompanied by the degradation of substrate proteins of effector caspases, including PARP, as evidence that caspase-dependent apoptosis was induced (Kiraz et al., 2016[36]; Hassan et al., 2014[23]). The activation of caspase cascade is tightly regulated by the expression of various regulators. Among them, Bcl-2 family members, which consist of pro-apoptotic and anti-apoptotic proteins, play a key role in determining the progression of the intrinsic apoptosis pathway. As a representative pro-apoptotic protein, Bax, located on the outer mitochondrial membrane, induces cytochrome *c* release by promoting mitochondrial permeability transition, or weakening the barrier function of the mitochondria outer membrane. Conversely, anti-apoptotic proteins, such as Bcl-2, are essential for maintaining mitochondrial permeability and membrane barrier stabilization to inhibit the release of apoptogenic factors (Bock and Tait, 2020[3]; Popgeorgiev et al., 2018[47]). Therefore, the balance between the pro-apoptotic and anti-apoptotic Bcl-2 family proteins acts as a determinant that induces activation of the caspase cascade upon initiation of the intrinsic pathway. In the current study, the caspase-9 and caspase-3 activities and degradation of PARP were obviously increased, and the Bax/Bcl-2 expression ratio was also enhanced in H_2_O_2_-treated MC3T3-E1 cells, which are consistent with previous studies (Cheng et al., 2019[8]; Xia et al., 2017[58]; He et al., 2015[24]). As expected, these changes were all significantly reversed in the presence of FO, indicating that FO can protect MC3T3-E1 cell apoptosis by inhibiting the intrinsic apoptosis pathway activated by oxidative stress. However, the protective potential of FO was almost completely obstructed by the blocking of HO-1 expression by transfection with Nrf2 siRNA, implying that the enhanced expression of HO-1 is adaptively responsible for the protection of mitochondrial dysfunction by FO. These results well support previous findings that the activation of Nrf2/HO-1 signaling in osteoblasts could be recognized as a protective mechanism for initiating an intrinsic apoptosis pathway, following oxidative stress-mediated mitochondrial dysfunction (Jin et al., 2020[30]; Cheng et al., 2019[8]; Han et al., 2017[20]; Choi et al., 2016[9]).

## Conclusions

In the present study, we elucidated that FO activated Nrf2/HO-1 signaling pathway, to protect against oxidative stress-mediated DNA damage and apoptosis in MC3T3-E1 osteoblasts. According to our findings, FO reversed the increased intracellular ROS production and mitochondrial damage caused by H_2_O_2_, eventually inhibiting DNA damage and apoptosis. In this process, FO activated Nrf2 and promoted the expression of its downstream target protein HO-1, presumably alleviating oxidative stress, while enhancing oxidant resistance. These results provide evidence that FO may have high applicability as a therapeutic or dietary supplement for maintaining osteoblast function from oxidative stress. However, further studies are required to assess how FO can regulate the transcriptional activity of Nrf2, including evaluation in animal models, and whether other signaling pathways can intervene in the antioxidant activity of FO.

## Conflict of interest

The authors declare that there is no conflict of interest.

## Acknowledgement

This research was part of the project titled ‘Development of functional food products with natural materials derived from marine resources (20170285),’ funded by the Ministry of Oceans and Fisheries, Republic of Korea.
